# Milk Composition for Admixed Dairy Cattle in Tanzania

**DOI:** 10.3389/fgene.2018.00142

**Published:** 2018-04-24

**Authors:** Evans K. Cheruiyot, Rawlynce C. Bett, Joshua O. Amimo, Fidalis D. N. Mujibi

**Affiliations:** ^1^Department of Animal Production, College of Agriculture and Veterinary Sciences, University of Nairobi, Nairobi, Kenya; ^2^Nelson Mandela Africa Institution of Science and Technology, Arusha, Tanzania; ^3^USOMI Limited, Nairobi, Kenya

**Keywords:** milk composition, breed type, genetic group, genomic markers, SNP, crossbred cows, Tanzania

## Abstract

It is well established that milk composition is affected by the breed and genotype of a cow. The present study investigated the relationship between the proportion of exotic genes and milk composition in Tanzanian crossbred dairy cows. Milk samples were collected from 209 animals kept under smallholder production systems in Rungwe and Lushoto districts of Tanzania. The milk samples were analyzed for the content of components including fat, protein, casein, lactose, solids-not-fat (SNF), and the total solids (TS) through infrared spectroscopy using Milko-Scan FT1 analyzer (Foss Electric, Denmark). Hair samples for DNA analysis were collected from individual cows and breed composition determined using 150,000 single nucleotide polymorphism (SNP) markers. Cows were grouped into four genetic classes based on the proportion of exotic genes present: 25–49, 50–74, 75–84, and >84%, to mimic a backcross to indigenous zebu breed, F1, F2, and F3 crosses, respectively. The breed types were defined based on international commercial dairy breeds as follows: RG (Norwegian Red X Friesian, Norwegian Red X Guernsey, and Norwegian Red X Jersey crosses); RH (Norwegian Red X Holstein crosses); RZ (Norwegian Red X Zebu and Norwegian Red X N’Dama crosses); and ZR (Zebu X GIR, Zebu X Norwegian Red, and Zebu X Holstein crosses). Results obtained indicate low variation in milk composition traits between genetic groups and breed types. For all the milk traits except milk total protein and casein content, no significant differences (*p* < 0.05) were observed among genetic groups. Protein content was significantly (*p* < 0.05) higher for genetic group 75–84% at 3.4 ± 0.08% compared to 3.18 ± 0.07% for genetic group >84%. Casein content was significantly lower for genetic group >84% (2.98 ± 0.05%) compared to 3.18 ± 0.09 and 3.16 ± 0.06% for genetic group 25–49 and 75–84%, respectively (*p* < 0.05). There was no significant difference (*p* < 0.05) between breed types with respect to milk composition traits. These results suggest that selection of breed types to be used in smallholder systems need not pay much emphasis on milk quality differences as most admixed animals would have similar milk composition profiles. However, a larger sample size would be required to quantify any meaningful differences between groups.

## Introduction

Development of efficient strategies to optimize milk composition has long been an active area of research and continues to attract increasing interest for the global dairy industry. Milk component levels and characteristics are important factors that have a significant effect on dairy product quality and yield (Murphy et al., 2016). Farmers in many developed countries are currently paid for milk deliveries based on fat and protein levels (Bailey et al., 2005; Cunha et al., 2010) such that milk composition has taken new importance in the dairy industry having a direct impact on farmers’ income and product processing. As such, the dairy industry must make strategic decisions on optimizing factors that affect milk composition to better meet the ever-changing technological requirements and consumer preferences.

In East Africa, milk component pricing based on milk fat, true proteins, and other dairy solids has not been adopted. However, major dairy processors in the region have expressed strong interest in implementing a quality-based pricing system and routinely offer bonus payment depending on other measures of milk quality (which include both compositional completeness as well as somatic cell and bacterial counts; Foreman and De Leeuw, 2016). This has been largely driven by the demand for high-quality dairy products that meet consumer and export market demand. Kenya is currently the only country in Africa which has recently implemented a quality-based milk payment system (QBPS; Foreman and De Leeuw, 2016).

Whereas there are three broad options for modifying milk composition: (i) cow nutrition and management, (ii) cow’s genetic intervention, and (iii) dairy manufacturing technologies, long-term changes of milk parameters can be achieved through breeding and other genetic interventions(Walker et al., 2004).

Significant progress has been made in the past to improve the gross composition of milk through selective breeding and nutrition management of cows (Jenkins and McGuire, 2006). Bovine milk composition is influenced by many factors including breed and genotype (Coleman et al., 2010; Palladino et al., 2010; Gustavsson et al., 2014), nutrition (Welter et al., 2016), season (Heck et al., 2009), parity (Yang et al., 2013), stage of lactation (Stoop et al., 2009), as well as the physiological state of the animal (Gurmessa and Melaku, 2012) which offer many practical ways of altering milk composition. Previous studies have established the potential to exploit variation of milk composition among breeds to improve milk quality (Glantz et al., 2009; Heck, 2009).

According to De Marchi et al. (2008), the breed of the cow is the main genetic aspect affecting milk quality characteristics, cheese making technology, and quality of dairy products. Variations in the milk composition among breeds have been widely demonstrated in the literature (see review Schwendel et al., 2015). Although it is well established that there is significant variation in milk quality among cattle breeds, little is known about the variation in milk composition of different dairy crosses with varying admixture levels. The limited studies available have shown that increasing the proportion of exotic genes in a cow leads to decreased milk component levels (Haile et al., 2008; Islam et al., 2014).

In smallholder systems, pedigree records are typically unavailable. The only way to estimate an animals’ breed composition is by way of molecular markers and admixture analysis. The use of single nucleotide polymorphism (SNP) markers for prediction of breed composition of admixed animals is gaining popularity with the substantial decrease in genotyping costs. Kuehn et al. (2011) and Frkonja et al. (2012) have demonstrated accurate prediction of breed composition using SNP markers in admixed cattle populations. The information on breed composition obtained through SNP markers is not only useful in understanding the variation of milk traits in crossbred animals, but also allows their incorporation into genomic selection programs to improving milk quality traits (VanRaden and Cooper, 2015).

Crossbreeding of local indigenous breeds with exotic cattle has been widely adopted in Tanzania since independence, mainly with the aim of increasing the productivity of local breeds. Often, these breeding practices are carried out indiscriminately resulting in animals with unknown and large variation in breed composition (Weerasinghe et al., 2013). Therefore, the complex within herd genetic composition and variability in Tanzania provides a unique opportunity to investigate the effect of breed admixture on milk quality traits in a smallholder setting as well as under a wide range of production environments. Understanding the milk quality profile of crossbred cattle is critical in the planning for the extent to which smallholder farmers, who are the main suppliers of milk in East Africa, can participate and maximize their incomes in the QBPS.

The aim of this study was to evaluate the relationship between individual animal exotic gene proportions and associated milk composition profiles. In addition, the study examines the effect of breed types and other environmental factors on milk components.

## Materials and Methods

### Ethics Statement

This study was performed following the International Livestock Research Institute (ILRI) Institutional Animal Care and Use Committee (IACUC) guidelines, with approval reference number 2014.35. Animals were handled by experienced animal health professionals to minimize discomfort and injury.

### Site Selection and Animal Recruitment

The study was undertaken in two districts of Tanzania, namely, Rungwe and Lushoto located in the Southern Highlands and the Usambara Mountains in Tanga, respectively. Study sites were chosen based on the possible availability of a wide range of breeds, the density of improved dairy cattle, the presence of other dairy cattle projects led by ILRI under the “Maziwa Zaidi” platform, and the site having been identified as being in an emerging high dairy potential region in Tanzania.

Households selected to participate in the study were recruited based on strict entry criteria. They had to have at least two cows, one of which was in milk or have a crossbred bull in active service. Additionally, unrelated animals were preferred and where possible households with observable breed diversity were sought. Animal recruitment was purposive within households. To qualify, animals had to be either pregnant heifers or cows in the third trimester of pregnancy or a cow that had calved 3 months prior to recruitment.

### Hair Samples and Genotyping

Hair samples were collected from the tail switch of the animals, taking care to avoid fecal contamination following the protocol described by the Animal Genetics Laboratory (2013). A total of 839 samples were obtained from 490 animals in Rungwe district and 349 animals in Lushoto district. Samples were genotyped at Geneseek (Neogen Corporation, Lincoln, NE, United States) using the Geneseek Genomic Profiler (GGP) high-density (HD) SNP array consisting of 150,000 SNPs. Data quality control on the merged data (study and reference) was undertaken using PLINK v 1.9 (Purcell et al., 2007). Data quality control included removal of SNPs with less than 90% call rate, less than 5% minor allele frequency (MAF), and samples with more than 10% missing genotypes. A total of 4,324 SNPs were removed, leaving 129,971 SNPs available for analysis. The unsupervised model-based clustering method implemented by the program ADMIXTURE v. 1.3.0 (Alexander et al., 2009) was used to estimate the breed composition of individual animals.

### Genetic Groups and Breed Types

Cows were classified into four genetic groups based on the individual admixture profile and level of exotic dairy genes (the whole complement of genetic material derived from international commercial dairy breeds). The groups were defined as follows: 25–49% exotic level (*n* = 20), 50–74% exotic level (*n* = 64), 75–84% exotic level (*n* = 43), and cows with >84% exotic level (*n* = 81) to mimic a backcross to indigenous zebu breed, F1, F2, and F3 crosses, respectively. Two explanations informed this definition. First, the range indicated around the classic proportions (50, 75%, etc.) expected provides for possible outcomes of Mendelian sampling. Second, due to the need to balance the number of individuals in each genetic group, a hard cutoff point was not considered, e.g., instead of the F3 starting at 82.5%, we used >84% to ensure that a sufficient number of animals were available in the lower group. One individual cow had less than 25% exotic gene composition and was excluded from the study. Additionally, cows were categorized into four breed types according to the level of international commercial dairy breeds as follows: RG (Norwegian Red X Friesian, Norwegian Red X Guernsey, and Norwegian Red X Jersey); RH (Holstein X Norwegian Red and Norwegian Red X Holstein); RZ (Norwegian Red X Zebu and Norwegian Red X N’Dama); and ZR (Zebu X GIR, Zebu X Norwegian Red, and Zebu X Holstein). The first breed in the combinations is the dominant breed in terms of proportions of exotic genes present. This grouping resulted in 9, 51, 109, and 39 individuals for the RG, RH, RZ, and ZR types, respectively. Both genetic group and breed types were assigned to each cow using the admixture methodology.

### Cluster Analysis

The clusters used in this study were obtained from classification done as part of the larger AgriTT (Agricultural Technology Transfer) project (manuscript in preparation). Briefly, baseline data encompassing the totality of farm characteristics as well as farmer’s behavior and characteristics were subjected to cluster analysis to group households into production/management groups. Next, factor analysis was performed and five broad factors that can be used to describe smallholder farmers in the study sites were derived: supplementation intensity and diversity of supplement use, milk productivity and sale, use of maize germ and bran, household wealth, and the purchase and the intensity of use of Napier grass. These extracted factors were subjected to cluster analysis. The squared Euclidean distance and Ward’s linkage method were used as the criterion for determining inter-object distance. Duda and Hart’s index stopping rule was used to decide on the optimum number of clusters. The analysis revealed four distinct production clusters. The main factors that determined the production environment groupings were: the intensity of feed supplementation as well as the diversity of supplements used; the level of milk productivity and sales per cow; the off-farm income and the size of land owned; and the use of maize germ or maize bran and the extent of purchase of Napier grass, the main source of cultivated forage in the country. Households in cluster 1 (26%) were characterized by low production and sale of milk as well as low usage of maize germ supplements. Cluster 2 households (33%) were characterized by the intense use of supplements such as maize germ and oil by-products and higher milk production. Cluster 3 households (24%) were characterized by low intensity and diversity of supplement usage. Cluster 4 had households (17%) that predominantly used maize germ at high intensity as the main supplement with little diversity of other supplements. Given that the herd sizes were very small (some farmers had only two qualifying animals in the analysis), these production clusters served as the contemporary group used in the association analysis.

### Milk Samples

Approximately 10 ml of raw milk was collected in the months of June 2015 and December 2015 from each of 209 cows in both Rungwe and Lushoto districts. A larger sample size could not be obtained given that milk yields in the target households are often low and farmers would not agree to larger samples being drawn.

Sampling was done once per animal for either morning or evening milk. The samples collected were immediately put under ice and transported to a field lab for storage at -20°C until later transportation to the ILRI, Nairobi, for analysis. Transportation from the field labs to ILRI was done with the samples placed under dry ice.

Information regarding parity, the age of the cow, and season of calving for each cow was also collected. Other variables related to production system including farm characteristics, feeding practices, as well as general health management practices were recorded and used to determine production clusters. Since the cows in the study sites were managed differently, cluster analysis was necessary to group animals into homogeneous clusters in order to minimize the confounding effect of production management on milk component traits. The number of milk samples available for the present study from each cluster was 57, 90, 37, and 25 for cluster 1, 2, 3, and 4, respectively. Only one milk sample was available for each cow.

### Laboratory Analysis of Milk Composition

Milk samples were evaluated for the content of fat, protein, casein, lactose, solids-not-fat (SNF), as well as total solids (TS) content by infrared spectroscopy using Milko-Scan FT1 analyzer (Foss Electric, Denmark) at the ILRI, Nairobi, Kenya.

The Milko-Scan FT1 analyzer requires a minimum of 26 ml of milk for duplicate analysis of each sample. However, since the total milk sample volume obtainable was low (8–12 ml), samples had to be diluted to obtain the optimum volume suitable for analysis. Consequently, and before analysis, two dilution procedures were undertaken based on the exact volume of each milk sample. Samples with 10 ml volume were diluted to 33.3% (v/v) in distilled water to obtain 30 ml while samples with less than 10 ml were diluted to 16.7% (v/v).

### Statistical Analysis

To obtain regression models for predicting the actual milk composition for the diluted study samples, 50 ml fresh milk samples from 15 individual cows were collected from the University of Nairobi farm. The milk samples were collected purposely from crossbred cows to be comparable with the study cows with respect to genetic composition. The cows at the University of Nairobi farm are managed semi-intensively and were milked twice a day. Samples were analyzed immediately after collection using Milko-Scan FT1 analyzer (Foss Electric, Denmark). Three sets of estimates [undiluted milk, dilution 1 (33.3% v/v), and dilution 2 (16.7% v/v)] for milk component content were obtained for each sample.

After checking for normality and presence of outliers for each of the analyzed milk trait (fat, protein, casein, lactose, and SNF), two prediction models were obtained by regressing milk composition estimates for the undiluted milk samples on the diluted samples using the REG procedure of SAS version 9.2 (SAS Institute, Inc., 2008) to obtain two separate models for each dilution. Before analysis, the values obtained for fat percentage were log transformed to base 10 to correct for non-uniform variance and skewness. All the other milk components (protein, casein, lactose, SNF, and TS) did not show any obvious deviation from normality or non-constancy of variance, and hence they were not log transformed. Actual milk component content of the study cows was determined as predicted values using the defined models for the respective dilutions.

To find out the relationship between breed type and genetic group on predicted milk composition traits, data were analyzed using the MIXED procedure in SAS version 9.2. Fixed effects included in the model were the genetic group, breed type, the age of the cow (at the time of milk sample collection), the month of sampling, and production cluster membership of cows (cluster).

Component trait averages for each genetic group and the breed type were obtained by fitting two separate statistical models, Model 1 and Model 2 for breed types and genetic group, respectively.

Model 1: *Y_ijkl_* = *u* + breed-type*_i_* + age*_j_*+ month*_k_* + cluster*_l_* + *e_ijkl_*
Model 2: *Y_ijkl_* = *u* + genetic-group*_i_* + age*_j_* + month*_k_* + cluster*_l_* + *e_ijkl_*,

where *Y_ijkl_* = individual sample measurement of fat, protein, casein, lactose, SNF, or TS content; *u* = overall mean; breed-type*_i_* = fixed effect of breed-type *i* (*i* = RG, RH, RZ, and ZR); genetic-group*_i_* = fixed effect of genetic group*_i_* (*i* = 25–49% exotic level, 50–74% exotic level, 75–84% exotic level, and >84% exotic level); age*_j_* = fixed effect of the *j*th age in years (*j* = 2, 3, 4, 5–10, and >10); month*_k_* = fixed effects of the *k*th month of milk sample collection (*k* = June and December); cluster*_i_* = fixed effect of the *i*th cluster (*i* = 1, 2, 3, and 4); and *e_ijkl_* = random residual term ∼*N* (0, σ^2^e). The degrees of freedom were calculated according to the Satterthwaite method (DDFM = Satterth).

Although farmers provided parity information for study cows, this information was mainly based on guesses and estimates (since most farmers purchase cows that are already in production and have calved several times before). As such, parity information was deemed unreliable and was excluded from the analysis. The significance of the fixed effects included in the two models was tested using the F statistic (*p* < 0.05). For the main effects of genetic group and breed type, multiple comparisons of least square means were performed using Tukey test with significance set at *p* < 0.05.

## Results

### Summary Statistics

**Table 1** summarizes the number of animals per breed type, genetic group, and cluster included in the analysis. Most animals consisted of crosses of Norwegian Red and East African Shorthorn Zebu (RZ) breeds. Compared to other genetic groups, a relatively high proportion (39%) of cows were represented in the genetic group with greater than 84% exotic genes (>84%). On the other hand, the lowest proportion (10%) of animals was represented in the genetic group with 25–49% exotic genes. Overall, the differences between means were small for all traits, within breed types, genetic groups, and production clusters.

**Table 1:** Summary of the number of cows per breed type, genetic group, and production cluster included in the study and their respective raw means ± *SD* of each milk trait.

	Number of animals	Fat (%)	Protein (%)	Casein (%)	Lactose (%)	SNF (%)	TS (%)
**Breed type^1^**							
RG	9	3.56 ± 1.9	3.20 ± 0.6	2.78 ± 0.4	4.35 ± 0.2	7.69 ± 0.5	12.27 ± 2.3
RH	51	4.0 ± 1.5	3.26 ± 0.5	2.89 ± 0.4	4.21 ± 0.4	7.39 ± 0.9	11.68 ± 1.7
RZ	109	3.33 ± 1.5	3.26 ± 0.5	3.0 ± 0.4	4.32 ± 0.4	7.56 ± 0.8	11.59 ± 2.1
ZR	39	3.38 ± 1.2	3.18 ± 0.4	2.94 ± 0.4	4.22 ± 0.4	7.37 ± 0.8	11.56 ± 2.1
**Genetic group^2^**							
25–9%	20	3.33 ± 1.2	3.21 ± 0.4	3.00 ± 0.4	4.34 ± 0.4	7.62 ± 0.8	11.46 ± 1.4
50–74%	64	3.36 ± 1.5	3.23 ± 0.4	2.94 ± 0.4	4.25 ± 0.4	7.44 ± 0.7	11.75 ± 2.3
75–84%	43	3.50 ± 1.6	3.41 ± 0.5	3.12 ± 0.4	4.36 ± 0.4	7.66 ± 0.9	11.77 ± 2.0
>84%	81	3.68 ± 1.5	3.16 ± 0.5	2.84 ± 0.4	4.23 ± 0.4	7.39 ± 0.8	11.51 ± 1.8
**Cluster^3^**							
Cluster 1	57	4.33 ± 1.6	3.27 ± 0.4	2.89 ± 0.3	4.29 ± 0.4	7.42 ± 0.8	12.73 ± 2.0
Cluster 2	90	3.53 ± 1.5	3.22 ± 0.4	2.84 ± 0.4	4.22 ± 0.5	7.36 ± 0.8	11.51 ± 2.1
Cluster 3	37	2.83 ± 1.2	3.38 ± 0.6	3.31 ± 0.4	4.42 ± 0.3	7.93 ± 0.8	11.00 ± 1.3
Cluster 4	25	2.59 ± 1.1	3.04 ± 0.4	2.97 ± 0.4	4.23 ± 0.3	7.42 ± 0.6	10.47 ± 1.0

*^*1*^Breed types were classified based on the individual breed composition estimated from SNP markers: RG = (Norwegian Red X Frisian, Norwegian Red X Guernsey, and Norwegian Red X Jersey); RH = (Holstein X Norwegian Red and Norwegian Red X Holstein); RZ = (Norwegian Red X Zebu and Norwegian Red X N’Dama), and ZR = (Zebu X GIR, Zebu X Norwegian Red, and Zebu X Holstein).*

*^*2*^Proportion of exotic genes estimated using SNP genotype markers and classified into four classes as cows with 25–49% exotic genes, 50–74% exotic genes, 75–84% exotic genes, and those with greater than 84% exotic genes.*

*^*3*^Classification of households based on the farm characteristics; SNF, solids-not-fat; TS, total solids.*

### Effect of Milk Dilution on Parameter Estimates

Milk samples were diluted in order to obtain the volume required by the infrared spectrometer to quantify the content of the milk components. Regression equations were then used to determine the predicted component content of the undiluted milk samples. The prediction model’s coefficient of determination (*R*^2^), root-mean-square error (RMSE), and the coefficient of variation (CV) for the analyzed milk traits are presented in **Table 2**. The coefficients of determination of the prediction models for the milk traits ranged from 91 to 99%. The parameter estimates for all the milk traits were slightly lower for dilution 2 (16.7% v/v) compared to dilution 1 (33.3% v/v). Fat content exhibited the largest CV; 2.1 and 4.6 for dilution 1 and dilution 2, respectively (**Table 2**). On the other hand, lactose had the small relative variability (CV = 0.77 and 1.23 for dilution 1 and dilution 2, respectively).

**Table 2:** Coefficient of determination (*R*^2^), root-mean-square error (RMSE), and coefficient of variation (CV) of the prediction models for the milk traits derived from the University of Nairobi dairy cattle used as a training population.

Trait (%)	Dilution 1 (33.3% v/v)			Dilution 2 (16.7% v/v)		
	*R*^2^	RMSE	CV	*R*^2^	RMSE	CV
Fat	0.99	0.09	2.1	0.97	0.19	4.62
Protein	0.99	0.05	1.3	0.97	0.09	2.41
Casein	0.99	0.04	1.38	0.98	0.05	1.8
Lactose	0.97	0.04	0.77	0.94	0.06	1.23
SNF	0.94	0.09	1.06	0.91	0.11	1.23
TS	0.99	0.14	1.02	0.98	0.24	1.78

*SNF, solids-not-fat; TS, total solids.*

### Estimates for Milk Composition Traits

The descriptive statistics and CV for the analyzed milk traits are presented in **Table 3**. The mean contents for fat, protein, casein, lactose, SNF, and TS content were 3.70, 3.24, 2.95, 4.28, 7.49, and 11.64, respectively. Of all the milk traits, fat content and lactose had the largest (38.23%) and lowest (9.63%) CV, respectively. Milk total protein and casein displayed a relatively moderate and similar CV with mean content ranging from 2.24 to 4.78 for protein and 2.14 to 4.22 for casein.

**Table 3:** Means and the coefficients of variation of the predicted milk traits for the study samples (Tanzanian milk data).

Trait (%)	Mean	Minimum	Maximum	CV (%)
Fat	3.70	1.58	6.92	38.23
Protein	3.24	2.24	4.78	14.45
Casein	2.95	2.14	4.22	14.24
Lactose	4.28	2.72	5.11	9.63
SNF	7.49	4.84	9.86	10.56
TS	11.64	7.71	17.64	17.03

*CV, coefficient of variation; SNF, solids-not-fat; TS, total solids.*

### Effects of Genetic and Non-genetic Factors on Milk Constituents

#### Genetic Factors

A fixed model was used to determine the relationships between milk component content and a set of fixed effects. The fixed effects included in the model were breed type, dairyness (proportion of exotic genes), age of the cow, production cluster, and month of sampling.

##### Age of the cow

The least square means for the effect of the age of the cow are provided in **Table 4**. Overall, the age of the cow did not have significant effect on all the milk component traits (*p* < 0.05).

**Table 4:** Least square means and standard errors for milk component traits for the age of the cow.

Trait (%)	Age class of the cow (years)^1^			
	3	4	5–10	>10
	Mean ± *SE*	Mean ± *SE*	Mean ± *SE*	Mean ± *SE*
Fat	3.00 ± 0.5	3.55 ± 0.22	3.62 ± 0.13	2.95 ± 0.36
Protein	3.41 ± 0.15	3.23 ± 0.08	3.26 ± 0.04	3.20 ± 0.11
Casein	3.23 ± 0.12	3.04 ± 0.06	3.03 ± 0.03	3.01 ± 0.09
Lactose	4.49 ± 0.14	4.49 ± 0.07	4.33 ± 0.04	4.14 ± 0.10
SNF	7.75 ± 0.25	7.56 ± 0.13	7.60 ± 0.07	7.32 ± 0.19
TS	10.76 ± 0.62	11.72 ± 0.32	11.47 ± 0.18	10.61 ± 0.46

*^*1*^Age of the cows reported by farmers at the time of milk collection and defined in four classes (3, 4, 5–10, and >10 years).*

*SE, standard errors; SNF, solids-not-fat; TS, total solids.*

##### Genetic group

The least square means for the effect of genetic group are provided in **Table 5**. The genetic group of the cows had a significant effect on total protein and casein content (*p* < 0.05). The total protein content was higher (3.4 ± 0.08%) in the 75–84% genetic group compared to 3.18 ± 0.07% in the >84% genetic group. Similarly, casein content significantly (*p* < 0.05) differed in three genetic groups: 25–49, 75–84, and >84%, with the highest content observed for genetic group 25–49% (3.18 ± 0.1%) and the lowest for genetic group >84% (2.98 ± 0.05%). We observed no significant difference (*p* < 0.05) for fat, lactose, SNF, and TS between genetic groups.

**Table 5:** Least square means and standard errors for milk component traits for each genetic group.

Trait (%)	Genetic group^1^			
	25–49%	50–74%	75–84%	>84%
	(*n*^2^ = 20)	(*n* = 64)	(*n* = 43)	(*n* = 81)
	Mean ± *SE*^3^	Mean ± *SE*	Mean ± *SE*	Mean ± *SE*
Fat	2.73 ± 0.37	2.84 ± 0.22	3.08 ± 0.26	3.04 ± 0.22
Protein	3.24 ± 0.12	3.25 ± 0.07	3.4 ± 0.08^a^	3.18 ± 0.07^b^
Casein	3.18 ± 0.09^a^	3.09 ± 0.06	3.16 ± 0.06^a^	2.98 ± 0.05^b^
Lactose	4.32 ± 0.11	4.25 ± 0.06	4.32 ± 0.07	4.22 ± 0.06
SNF	7.72 ± 0.2	7.54 ± 0.12	7.64 ± 0.13	7.5 ± 0.11
TS	10.92 ± 0.49	11.35 ± 0.3	11.35 ± 0.32	10.87 ± 0.28

*^*1*^Proportion of exotic genes estimated using SNP genotype markers and classified into four classes as cows with 25–49% exotic genes, 50–74% exotic genes, 75–84% exotic genes, and those with greater than 84% exotic genes.*

*^*2*^Number of samples in each genetic group.*

*^*3*^Standard errors.*

*SNF, solids-not-fat; TS, total solids.*

*^*a,b*^Means within a row with different superscripts differ significantly (*p* < 0.05).*

**FIGURE 1:**
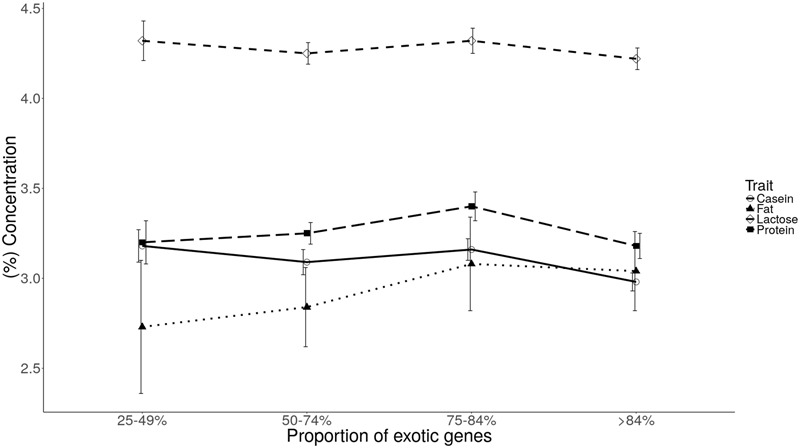
Least square means of milk fat, protein, casein, and lactose for each level of exotic genes.

**FIGURE 2:**
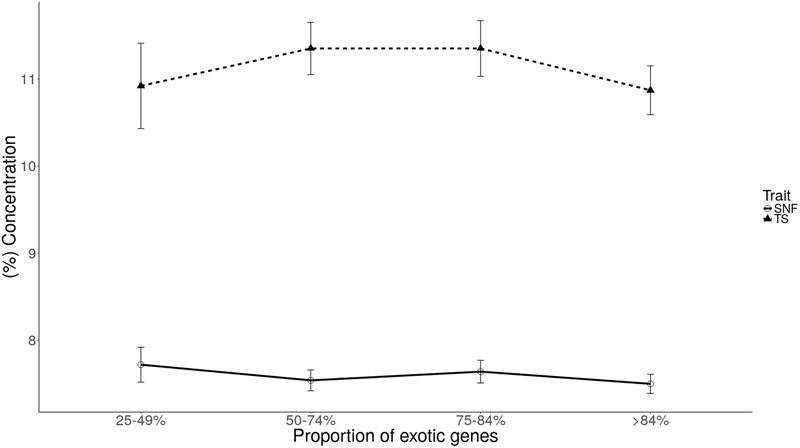
Least square means of solids-not-fat (SNF) and total solids (TS) for each level of exotic genes.

Plots of least square mean estimates for milk component traits by genetic group are shown in **Figures 1, 2**. Overall, the mean was higher for the 75–84% genetic group and lowest for the 25–49% genetic group. For fat and protein, the trend seems to suggest a general increase in component levels as dairyness increases, with a sharp drop for the animals in the >84% group. For lactose and casein, the trend is not clear. However, the drop for the >84% group is consistent for all components evaluated.

##### Breed type

**Table 6** gives the least square means and associated standard errors of the milk traits for each breed type. Overall, we observed no significant difference in milk composition among breed types (*p* < 0.05). The RG breed type (consisting of crossbreeds of Jersey, Guernsey, Holstein, and Norwegian Red breed) had the highest average fat content (4.05 ± 0.51%) while breed type ZR (consisting of crossbreeds of Zebu and Norwegian Red breed) had the lowest average fat content (3.04 ± 0.25%). The total protein and casein content was similar across breed types.

**Table 6:** Least square means and standard errors for milk component traits per breed type.

Trait (%)	Breed type^1^			
	RG	RH	RZ	ZR
	Mean ± *SE*	Mean ± *SE*	Mean ± *SE*	Mean ± *SE*
Fat	4.05 ± 0.51	3.48 ± 0.23	3.21 ± 0.19	3.04 ± 0.25
Protein	3.21 ± 0.17	3.28 ± 0.08	3.28 ± 0.06	3.23 ± 0.09
Casein	2.95 ± 0.13	3.0 ± 0.07	3.11 ± 0.05	3.12 ± 0.08
Lactose	4.37 ± 0.14	4.2 ± 0.07	4.32 ± 0.05	4.26 ± 0.08
SNF^3^	7.83 ± 0.10	7.46 ± 0.13	7.66 ± 0.10	7.55 ± 0.15
TS^4^	11.17 ± 0.29	11.35 ± 0.33	11.06 ± 0.25	11.12 ± 0.38

*^*1*^Breed types were classified based on the individual breed composition estimated from SNP markers: RG = (Norwegian Red X Frisian, Norwegian Red X Guernsey, and Norwegian Red X Jersey); RH = (Holstein X Norwegian Red and Norwegian Red X Holstein); RZ = (Norwegian Red X Zebu and Norwegian Red X N’Dama); and ZR = (Zebu X GIR, Zebu X Norwegian Red, and Zebu X Holstein).*

*SE, standard errors; SNF, solids-not-fat; TS, total solids.*

#### Non-genetic Factors

##### Effect of season

In this study, the months of sampling coincided with the two seasons in Tanzania: wet season (June) and dry season (December). Least square means and the respective *SD* for the effect of season on milk traits are given in **Table 7**. The month of sampling had a significant effect (*p* < 0.001) on the content of milk fat, casein, and SNF. Casein content was higher in milk sampled in the wet season (3.27 ± 0.06%) than in the dry season (2.88 ± 0.06%) with a mean difference of 0.39 ± 0.08%. Similarly, SNF was greater in the wet season (7.81 ± 0.13%) than in the dry season (7.32 ± 0.12%) with a recorded mean difference of 0.49 ± 0.18%. On the contrary, fat content was significantly (*p* < 0.001) higher (3.97 ± 0.24%) in the dry season than in the wet season (2.59 ± 0.24%). The mean difference for the fat content was 1.38 ± 0.34%. The TS and lactose contents were not affected by the month of sampling (*p* = 0.089).

**Table 7:** Least square means and standard errors for milk component traits for month of sampling.

Trait (%)	Month of sampling	
	June	December
Fat	2.59 ± 0.24	3.97 ± 0.24
Protein	3.32 ± 0.08^a^	3.23 ± 0.07^b^
Casein	3.27 ± 0.06^a^	2.88 ± 0.06^b^
Lactose	4.34 ± 0.07	4.19 ± 0.06
SNF	7.81 ± 0.13^a^	7.32 ± 0.12^b^
TS	10.75 ± 0.3	11.54 ± 0.3

*SNF, solids-not-fat; TS, total solids.*

*^*a,b*^Means within a row with different superscripts differ significantly (*p* < 0.05).*

##### Effect of production environment

The least square means of the clusters is shown in **Table 8**. Cluster membership of cows significantly (*p* < 0.05) affected the total protein, casein, SNF, and TS content. Casein content was higher for cows in cluster 3 (3.23 ± 0.08%) and cluster 4 (2.91 ± 0.08%) (*p* = 0.0001 and *p* = 0.021, respectively). On the other hand, protein content was significantly lower in cluster 4 (3.05 ± 0.1%) compared to cluster 1 (3.35 ± 0.09%), cluster 2 (3.32 ± 0.08%), and cluster 3 (3.38 ± 0.1%). SNF content was higher in cluster 1 (7.62 ± 0.15%) than cluster 4 (7.28 ± 0.17%). TS content was significantly (*p* < 0.05) greater in cluster 1 (12.10 ± 0.35%) than in cluster 2 (11.0 ± 0.39%), cluster 3 (11.35 ± 0.32%), and cluster 3 (10.51 ± 0.42%). There was no significant difference in lactose and fat content among the clusters.

**Table 8:** Least square means and standard errors for milk component traits for each cluster.

Trait (%)	Clusters^1^			
	Cluster 1 (*n*^2^ = 57)	Cluster 2 (*n* = 90)	Cluster 3 (*n* = 37)	Cluster 4 (*n* = 25)
	Mean ± *SE*^3^	Mean ± *SE*	Mean ± *SE*	Mean ± *SE*
Fat	3.47 ± 0.26	3.07 ± 0.23	3.43 ± 0.32	3.16 ± 0.31
Protein	3.35^a^ ± 0.09	3.32 ± 0.08^a^	3.38 ± 0.1^a^	3.05 ± 0.1^b^
Casein	3.11 ± 0.07	3.06 ± 0.06	3.23 ± 0.08^a^	2.91 ± 0.08^b^
Lactose	4.31 ± 0.08	4.25 ± 0.06	4.34 ± 0.09	4.16 ± 0.09
SNF	7.62 ± 0.15^a^	7.57 ± 0.13	7.77 ± 0.16	7.28 ± 0.17^b^
TS	12.10 ± 0.35^a^	11.0 ± 0.39^b^	11.35 ± 0.32^b^	10.51 ± 0.42^b^

*^*1*^Groups of households defined based on the farm characteristics.*

*^*2*^Number of samples in each cluster.*

*^*3*^Standard errors.*

*SNF, solids-not-fat; TS, total solids.*

*^*a, b*^Means within a row with different superscripts differ significantly (*p* < 0.05).*

## Discussion

### Summary Statistics and Parameter Estimates

The small differences between means for all traits, within breed types, genetic groups, and production clusters observed in this study (**Table 1**) are likely because of small sample sizes within each grouping. Differences in the parameter estimates among the two dilutions used for prediction suggest a noticeable effect on the variability of milk composition and that the relationship between dilutions is not linear. Fat content, for instance, exhibited the largest CV; 2.1 and 4.6 for dilution 1 and dilution 2, respectively (**Table 2**). This variability may be partly attributed to the effect of the stability of milk fat emulsion and the varying sizes of fat globules (Suranindyah and Pretiwi, 2015) which probably becomes more unstable with increased dilution. On the other hand, the small relative variability of lactose (0.77 and 1.23 for dilution 1 and dilution 2, respectively) largely reflects its greater solubility in water. We undertook to collect milk samples for prediction specifically from crossbred cows at the University of Nairobi farm in order to be comparable to the study samples. However, it is important to point out that the milk samples obtained from the farm were from one herd and collected in the same season. On the contrary, the milk samples from Tanzania cows were collected over two seasons and from different management systems. The prediction equations obtained from dilution of samples from the University of Nairobi farm were useful because they provided a mechanism to understand how dilution affects milk component content and the resultant equations could then be used to predict the milk component content for the undiluted target samples. To the extent that the training data for producing the equations was only from a small sample set, the estimates for undiluted components could have introduced some bias.

### Estimates for Milk Composition Traits

Overall, the average milk component content recorded in this study was within the range of values reported in previous studies for Holstein–Friesian dairy breeds (Glantz et al., 2009; Palladino et al., 2010; Penasa et al., 2014). This is not surprising given that our analysis of admixture and genetic composition of the study population indicated a dominant Holstein–Friesian origin. Compared to studies by Heck et al. (2009) and Penasa et al. (2014), this study had larger CVs. However, results similar to those obtained in this study were reported by Varotto et al. (2015) except for fat content whose CV was much higher (38.23%) in the present study. It should be emphasized that the results observed in this study are predicted mean values obtained from the diluted milk samples. The large CV for the content of milk fat might be partly due to the effect of dilution.

### Effects of Genetic and Non-genetic Factors on Milk Constituents

#### Genetic Factors

##### Age of cow

Results indicated that the age of cow did not have a statistically significant effect on all milk component traits. This observation was expected given that the age information provided by the farmers was based on estimates rather than written records since most farmers purchase mature cows already in production, with no accompanying pedigree or performance records.

##### Genetic group

Previous studies have demonstrated the relationship between breed type and milk quality (Carroll et al., 2006; Palladino et al., 2010). However, smallholder production systems in sub-Saharan Africa utilize non-descript crossbred animals with unknown breed type. We used admixture analysis to estimate the breed proportions of known dairy breeds in the study cattle. Based on the extent of the dairyness (proportion of exotic genes) of the animals, they were grouped into four genetic groups as follows; 25–49, 50–74, 75–84, and >84%, to mimic a backcross, F1, F2, and F3 exotic crosses, respectively.

Although it is well documented that the genotype of the cow has significant effect on milk composition (Coleman et al., 2010; Schwendel et al., 2015), failure to detect any relationship between the genetic group and majority of the milk component traits studied (fat, lactose, SNF, and TS) could be related to the fact that our milk samples were obtained from smallholder farms characterized by diverse dairy management practices. These estimates are therefore confounded by other environmental influences acting on this genotype and which cannot be accounted for in our model, especially owing to the very small herd sizes. Additionally, the breed composition of the cow was based on admixture from many different breeds, which also adds to the complexity of estimating genetic effects. To understand the lack of significant difference between observed means, we performed a *post hoc* power analysis which revealed that a sample size of 580 was required to observe a detectable deference for an effect size of 0.23 (the difference observed between fat content for genetic group 25–49 and >84%), considering a power of 0.8 and 95% significance level. This was well beyond the available sample size (101 cows in the two genetic groups being compared) and reinforces the need for a larger study with appropriate sample size.

The trends for fat percentage observed here are quite contrary to expectations, since indigenous animals tend to have milk with higher fat percentage (Haile et al., 2008). Available data from literature indicate that average milk fat content ranges between 2.0 and 6.1% in animals fed total mixed ration (TMR) (Kelsey et al., 2003) and between 2.68 and 4.50% for grass-fed cattle (Kay et al., 2005; Myburgh et al., 2012). Our results fall within the range for grass fed cattle as expected given that most animals are subsist on leafy greens (mostly Guatemala or elephant/Napier grass) as the main feed. Additionally, it is well established that there are breed differences with respect to milk fat content (reviewed by Samková et al., 2012). Further, indicine cattle tend to have higher fat content than taurine cattle (Haile et al., 2008). Based on this premise, we expected that animals with relatively high indicine proportions (25–49% genetic group) would have higher milk fat composition. The disparity between our expectations and what was observed is likely due to a management effect, where the animals are kept in confinement but receive little supplemental feeds and thrive only on leafy greens whenever available. There are limited published studies of equivalent systems and animal types to compare our results to. In their study, Haile et al. (2008) using crosses of Holstein–Friesian and Boran reported that the content of milk components decreased with increasing the proportion of exotic gene content. This runs contrary to our results. These results could be due to the differences in relative sizes of the additive and heterosis effects which likely differed among genetic groups (Cunnigham and Syrstad, 1987). From our results, it would appear that the 75–84% genetic group maximizes the heterotic effects obtainable from the crossbred population studied.

##### Breed type

We found no significant difference in milk components content among breed types. However, the relatively higher fat percentage in breed type RG is probably due to the excess of Jersey and Guernsey genes in this breed type which is in conformity with numerous studies that indicate superior milk quality due to Jersey and Guernsey genes (Croissant et al., 2007; De Marchi et al., 2008; Palladino et al., 2010). The lack of variability in mean estimates among breeds is likely a function of our definition of breed types (as a combination of the breeds making up the top 75% gene composition in animal cow, with the breed name being defined by the breed of highest presence). It is also possible that an increase in sample size would allow the confounding effects to average out such that true differences can be estimated.

#### Non-genetic Factors

##### Effect of season

In Tanzania, there is an extreme seasonal fluctuation in milk production due to changes in rainfall and feed production for dairy animals (Nell et al., 2014). The seasonal variation of milk component levels observed in this study can be explained by seasonal changes in the composition of the feeds available to the animals. Jenkins and McGuire (2006) observed that lactose content in milk is less sensitive to dietary changes. The findings of this study are similar to those of other studies such as Auldist et al. (2000) and Heck et al. (2009) who also observed large seasonal variation of major composition in Holstein dairy cows. The higher fat content in the dry season compared to the wet season is likely related to reduced moisture levels in feeds as well as the feeding practices adopted. Typically, dairy feeding in smallholder system is largely based on crop residues, roadside grazing, and occasionally on fodder crops. However, the dry season in Tanzania is usually characterized by scarcity and poor quality of feeds. Farmers, therefore, tend to increase the use of commercial supplements such as oil by-products, maize germ, cottonseed cake, and sunflower cake. Nevertheless, the use of these concentrates has been shown to result in an increase in the content of milk fat (Carroll et al., 2006).

##### Effect of production environment

As described in the previous section, one of the key factors used for defining clusters in this study was the animal feeding practices adopted by smallholder farmers in the study sites. It is well established that diet has a profound effect on both milk composition and yield (Turner et al., 2006). Carroll et al. (2006) demonstrated that casein proportion decreases linearly with increased supplemental fat. It is not surprising, therefore, that cluster 4 characterized by intensive use of maize germ as supplement had lower casein content compared to cluster 3 which was characterized by the low intensity in the use of supplements. It has been proposed that increased use of concentrate supplements leads to decreased release of somatotropin which reduces mammary extraction of amino acids (Cant et al., 1993) and thus a decline in casein content.

Compared to cluster 1, cows in cluster 2 were managed intensively with diverse use of supplements such as maize bran and oilseed by-products. Notably, the farmers in cluster 1 practiced subsistence dairy farming, characterized by minimal supplementation that manifested as low productivity and low milk sales. Given the negative correlation between milk yield and TS (Bobe et al., 2007), the low milk yield and high TS content were expected. Based on the results of this study, it would appear that cluster 1 and cluster 3 maximize the milk component content of the study population.

## Conclusion

The results obtained in this study indicate low variability in milk composition traits among breed types and genetic groups (defined by the level of the exotic genes). The 75–84% genetic group tended to have superior performance with regard to maximizing milk component content. However, it is clear that a more rigorous and larger study would be required to understand how breed type and genetic group affect milk quality in systems with highly admixed animals. Such an understanding is critical in recommending the types of crossbred cows farmers need to keep in order to produce milk that meets market demand. Additionally, these results will be valuable in assessing the viability of an offtaker payment scheme based on the quality of milk delivered by farmers.

## Author Contributions

FM conceived, designed, and obtained funding for the study. EC performed the experiment. EC, FM, and JA analyzed the data. EC and FM drafted the manuscript. RB and JA made suggestions and corrections. All authors read and approved the final manuscript.

## Conflict of Interest Statement

EC and FM are currently employees of USOMI LTD. However, they completed the work detailed in the submitted manuscript before coming into said employment. The other authors declare that the research was conducted in the absence of any commercial or financial relationships that could be construed as a potential conflict of interest. The reviewer NK and handling Editor declared their shared affiliation.
